# Efficacy and survival prognosis analysis of surgical resection combined with targeted therapy in patients with colorectal cancer liver metastasis

**DOI:** 10.3389/fonc.2025.1692800

**Published:** 2025-11-10

**Authors:** Feihu Yan, Yunjie Shi, Zhengchun Kang, Hantao Wang, Xu Li

**Affiliations:** Department of Colorectal Surgery, The First Affiliated Hospital of Naval Medical University, Shanghai, China

**Keywords:** colorectal cancer, liver metastasis, surgical resection, targeted therapy, survival rate, prognosis

## Abstract

**Introduction:**

Colorectal cancer liver metastasis (CRLM) is a leading cause of death in colorectal cancer patients. Simple surgical resection has a high recurrence rate, and combining targeted therapy offers a new way to improve prognosis. Currently, the efficacy of surgery combined with targeted therapy and the influencing factors of prognosis still require in-depth exploration.

**Methods:**

From January 2019 to February 2022, 76 CRLM patients were randomly split into an observation group (n=38, surgery + chemotherapy + bevacizumab-based targeted therapy) and a control group (n=38, surgery + conventional chemotherapy). Key indicators were compared, and Cox regression analyzed prognosis factors.

**Results:**

There were no significant differences in operation time (185.6±32.4 min vs. 178.9±29.5 min) or intraoperative blood loss (210.3±56.7 ml vs. 205.8±51.2 ml) between groups (P>0.05). However, the observation group had a shorter hospital stay (10.2±2.1 days vs. 12.5±2.6 days, P<0.05), higher ORR (68.9% vs. 46.7%) and DCR (91.1% vs. 75.6%, both P<0.05), and better 1-, 2-, 3-year PFS (72.2%/45.6%/31.1% vs. 51.1%/26.7%/15.6%) and OS (86.7%/64.4%/48.9% vs. 71.1%/42.2%/27.8%, all P<0.05). The observation group also had a higher hypertension rate (23.3% vs. 6.7%, P<0.05), with no other significant adverse reaction differences (P>0.05). Cox regression showed targeted therapy and ≤3 liver metastases were independent factors for favorable prognosis (P<0.05).

**Discussion:**

Surgical resection combined with targeted therapy can effectively improve tumor control efficacy and long-term survival outcomes of CRLM patients, and shorten the hospital stay. Although this combined regimen increases the risk of hypertension, its overall safety is controllable.

## 1 Introduction

Colorectal cancer (CRC) is a malignant tumor with high incidence worldwide, and it imposes a heavy disease burden. Globally, there are over 2 million new CRC cases and more than 1 million CRC-related deaths each year. CRC accounts for 25% of deaths from digestive system malignant tumors (1). Early screening helps patients with stage I CRC achieve a 5-year survival rate of over 90%. However, about 20%-30% of patients have distant metastasis at the initial diagnosis. During the disease progression, the proportion of metastasis reaches as high as 50% (2, 3). The liver is the main target organ for hematogenous metastasis of CRC (4). Colorectal cancer liver metastasis (CRLM) is the main cause of death in CRC patients. Patients without active treatment have a median survival of only 6–9 months, and their 5-year survival rate is less than 5% (5). After R0 resection, the 5-year survival rate increases to 30%-50% (6). But the 1-year recurrence rate is 55%-60%, and the 3-year cumulative recurrence rate exceeds 75% (7).

Traditional chemotherapy regimens (such as FOLFOX and FOLFIRI) have an objective response rate (ORR) of only 30%-40% (8). Moreover, 15% of patients cannot complete the treatment course due to adverse reactions (9). Bevacizumab, a vascular endothelial growth factor (VEGF) inhibitor, can prolong progression-free survival (PFS) and overall survival (OS). It has become a first-line treatment option for CRLM (10). There are controversies regarding combined treatment for CRLM. Supporters argue that preoperative use of targeted therapy can improve the resection rate. They also believe that postoperative maintenance therapy can reduce the recurrence risk by 12%-15% (11). Opponents claim that combined treatment may increase the risk of intraoperative massive bleeding by 8% and the incidence of anastomotic leakage (12). Furthermore, existing studies have inconsistent conclusions. This is due to differences in sample sizes and treatment protocols (13, 14).

The innovations of this study are as follows: first, it strictly controls the interval between targeted drug use and surgery to be no less than 4 weeks to ensure safety; second, it focuses on the difference in efficacy among patients with no more than 3 metastatic lesions; third, it adopts standardized detection and follow-up procedures. This study aims to clarify the clinical value of combined treatment and provide a basis for individualized treatment of CRLM patients.

## 2 Research methods

### 2.1 Research subjects

This study adopted a randomized controlled design. A total of 76 patients with CRLM admitted from January 2019 to February 2022 were selected as the research subjects. The study protocol strictly followed the principles of the Declaration of Helsinki and was approved by the Hospital Ethics Committee (No. 2019012). All patients or their legal representatives signed written informed consent forms.

Patients were included in the study if they met the following criteria: a confirmed diagnosis of CRC via histopathological examination, with liver metastases verified by contrast-enhanced liver computed tomography (CT), magnetic resonance imaging (MRI), or intraoperative exploration; age ranging from 18 to 75 years and a Karnofsky Performance Status (KPS) score of ≥ 70; liver metastases that met the criteria for radical resection (R0 resection, defined as a resection margin of ≥ 1 cm from the tumor edge or pathological confirmation of no tumor cells at the margin); no other distant metastases (e.g., to the lung, bone, or brain); an estimated survival time of ≥ 3 months; basically normal function of major organs (including the liver, kidney, and heart) as well as blood routine and coagulation function indicators within the normal range (with platelets ≥ 100×10^9^/L, hemoglobin ≥ 90 g/L, and international normalized ratio ≤ 1.5); and no previous surgical treatment for liver metastases or targeted drug therapy.

Patients were excluded if they had any of the following conditions: comorbidity with other primary malignant tumors; allergy to bevacizumab or components of chemotherapeutic drugs; presence of uncontrolled hypertension (defined as a systolic blood pressure of ≥ 160 mmHg or a diastolic blood pressure of ≥ 100 mmHg), severe bleeding tendency (such as coagulation disorders), or active peptic ulcer; comorbidity with severe infection, metabolic diseases (e.g., uncontrolled diabetes mellitus), or mental illnesses; or incomplete clinical data or inability to cooperate with follow-up procedures.

#### 2.1.1 Sample size calculation

The primary outcome measure of this study was the 3-year post-operative overall survival (OS), and the sample size was estimated using PASS 15.0 statistical software. The calculation assumptions were based on previous study data: for patients with CRLM who received surgery combined with conventional adjuvant chemotherapy, the 3-year OS was approximately 25%-30%, while it was expected that the 3-year OS of the observation group (treated with surgery combined with adjuvant targeted therapy) would increase by 20%, reaching 47%-50%. The statistical parameters were set as follows: the α value (type I error rate) was 0.05 (two-sided), and the β value (type II error rate) was 0.2, which corresponded to a test power (1-β) of 80%. According to the sample size formula for comparing survival rates between two groups, the calculation results showed that each group required at least 34 patients. Considering a 20% dropout rate, 38 patients were finally determined to be included in each group, resulting in a total sample size of 76 patients—this was to ensure the study had sufficient statistical power to detect the expected difference in treatment efficacy.

#### 2.1.2 Randomization method

A random number table was generated using SPSS 26.0 statistical software, and 76 eligible patients were randomly divided into the observation group and the control group at a 1:1 ratio, with 38 patients in each group. The specific steps were carried out as follows: first, each patient was numbered sequentially from 1 to 76 based on the order of inclusion; then, the “Random Number Generation” function of SPSS 26.0 was used to produce 76 random numbers ranging from 0 to 1, and these random numbers were sorted by their values; finally, the first 38 patients after sorting were assigned to the observation group, while the last 38 patients were allocated to the control group. The randomization process was independently supervised by a statistician not involved in patient enrollment or treatment implementation to ensure allocation concealment.

The primary outcome measure of this study was the 3-year post-operative OS, and the sample size was estimated using PASS 15.0 statistical software. The calculation assumptions were based on previous study data: for patients with CRLM who received surgery combined with conventional chemotherapy, the 3-year OS was approximately 25%-30%, while it was expected that the 3-year OS of the observation group (treated with surgery combined with targeted therapy) would increase by 20%, reaching 47%-50%. The statistical parameters were set as follows: the α value (type I error rate) was 0.05 (two-sided), and the β value (type II error rate) was 0.2, which corresponded to a test power (1-β) of 80%. According to the sample size formula for comparing survival rates between two groups, the calculation results showed that each group required at least 34 patients. Considering a 20% dropout rate, 38 patients were finally determined to be included in each group, resulting in a total sample size of 76 patients—this was to ensure the study had sufficient statistical power to detect the expected difference in treatment efficacy.

#### 2.1.3 Secondary outcome measures

In addition to the primary outcome (3-year post-operative OS), secondary outcome measures included: 1) surgical and post-operative recovery indicators (operation time, intraoperative blood loss, length of hospital stay); 2) tumor efficacy evaluation indicators (objective response rate [ORR], disease control rate [DCR]) assessed 3 months after surgery per RECIST 1.1; 3) survival prognosis indicators (1-year, 2-year post-operative OS, 1-year, 2-year, 3-year post-operative progression-free survival [PFS], median PFS, median OS); 4) incidence of adverse events (graded per CTCAE 5.0) and post-operative recurrence site distribution; 5) identification of independent prognostic factors for CRLM via Cox regression analysis.

A random number table was generated using SPSS 26.0 statistical software, and 76 eligible patients were randomly divided into the observation group and the control group at a 1:1 ratio, with 38 patients in each group. The specific steps were carried out as follows: first, each patient was numbered sequentially from 1 to 76 based on the order of inclusion; then, the “Random Number Generation” function of SPSS 26.0 was used to produce 76 random numbers ranging from 0 to 1, and these random numbers were sorted by their values; finally, the first 38 patients after sorting were assigned to the observation group, while the last 38 patients were allocated to the control group.

### 2.2 Treatment methods

#### 2.2.1 Control group (surgery + conventional adjuvant chemotherapy)

Chemotherapy with the FOLFOX6 regimen was initiated 1 week after surgery. This timing was determined based on the study’s institutional protocol for rapid adjuvant intervention, but it should be noted that it is earlier than the 4–8 weeks recommended by most guidelines (e.g., NCCN, ESMO) to balance microscopic residual disease control with post-operative recovery. The specific regimen was as follows: Oxaliplatin (Sanofi Pharmaceuticals, Approval No.: National Drug Code H20000337) at 85 mg/m², intravenous infusion for 2 hours on Day 1; Calcium Folinate (Jiangsu Hengrui Medicine, Approval No.: National Drug Code H20000584) at 400 mg/m², intravenous infusion for 2 hours on Day 1; Fluorouracil (Shanghai Xudong Haipu Pharmaceutical, Approval No.: National Drug Code H31020593) at 400 mg/m² via intravenous bolus, followed by continuous infusion of 2400 mg/m² for 46 hours. The treatment was administered every 2 weeks as one cycle, with a total of 12 cycles.

#### 2.2.2 Observation group (surgery + adjuvant chemotherapy + adjuvant targeted therapy)

Targeted therapy was initiated on the same day as the first cycle of adjuvant chemotherapy (1 week after surgery), using bevacizumab. Similar to adjuvant chemotherapy, this 1-week post-operative start time is earlier than guideline-recommended intervals (4–8 weeks) and was chosen to maximize suppression of minimal residual disease. However, this may limit external validity, as many centers (especially in Western countries) typically delay adjuvant targeted therapy to avoid potential risks of wound dehiscence or delayed healing. The dose of bevacizumab was 5 mg/kg, which was diluted in 100 ml of 0.9% sodium chloride injection for intravenous infusion. The infusion time was ≥ 30 minutes, and there was a 1-hour interval between the infusion of bevacizumab and chemotherapeutic drugs. Targeted therapy was administered once every 2 weeks, synchronized with chemotherapy, for a total of 12 cycles.

### 2.3 Observation indicators and detection methods

Surgical and post-operative recovery indicators included operation time, intraoperative blood loss, and length of hospital stay. Operation time was recorded as the total duration from skin incision to the end of skin suture after surgery, accurate to minutes. Intraoperative blood loss was determined by the sum of blood collected by the aspirator and blood absorbed by gauze (1 dry gauze was calculated to absorb 5 ml of blood), accurate to milliliters. Length of hospital stay was the total number of days from the surgery day to the day when the patient met discharge criteria (stable vital signs, good wound healing, no obvious complications), accurate to days.

Efficacy was evaluated 3 months after surgery using the Response Evaluation Criteria in Solid Tumors (RECIST 1.1).

Complete Response (CR): All target lesions disappeared and persisted for more than 4 weeks;

Partial Response (PR): The sum of the diameters of target lesions decreased by ≥30% and persisted for more than 4 weeks;

Stable Disease (SD): The sum of the diameters of target lesions did not decrease to meet PR or increase to meet progression;

Progressive Disease (PD): The sum of the diameters of target lesions increased by ≥20%, or new lesions appeared.

ORR and Disease Control Rate (DCR) were calculated. ORR = (number of CR + PR cases)/total number of cases × 100%, and DCR = (number of CR + PR + SD cases)/total number of cases × 100%.

Survival prognosis indicators included PFS and OS. PFS was the time from the surgery date to the first occurrence of disease progression (imaging-confirmed increase in metastases or new lesions) or death from any cause. OS was the time from the surgery date to death from any cause.

Follow-up was conducted through a combination of outpatient visits and telephone calls. Patients were followed up once a month in the first 6 months after surgery, and once every 3 months after 6 months. Follow-up content included: 1) physical examination; 2) detection of serum CEA (electrochemiluminescence method, lower detection limit 0.5 ng/ml) and CA19-9 (chemiluminescence method, lower detection limit 0.6 U/ml); 3) contrast-enhanced liver MRI (1.5T, slice thickness 5 mm) at 6, 12, 24, and 36 months after surgery, and contrast-enhanced liver CT (slice thickness 5 mm) at other time points to confirm progression or recurrence. The follow-up deadline was February 2025. Post-operative 1-year, 2-year, and 3-year PFS and OS were calculated.

Adverse events were recorded in accordance with the Common Terminology Criteria for Adverse Events (CTCAE 5.0). The incidence of each adverse event was calculated.

Hypertension: Systolic blood pressure ≥140 mmHg or diastolic blood pressure ≥90 mmHg, or an increase of ≥30/20 mmHg compared with baseline blood pressure;

Proteinuria: Qualitative urine protein ≥++ or 24-hour quantitative urine protein ≥1 g;

Hand-foot syndrome: Manifested as numbness, paresthesia, erythema, swelling, pain, etc. in the palms or soles.

Factors potentially affecting prognosis were collected, including gender (male/female), age (<60 years/≥60 years), primary tumor location (colon/rectum), number of liver metastases (≤3/>3), pathological differentiation (well/moderately differentiated, poorly differentiated), receipt of targeted therapy (yes/no), and pre-operative CEA level (<5 ng/ml/≥5 ng/ml).

#### 2.3.1 Adverse event grading (supplemented granularity per CTCAE 5.0)

Adverse events were recorded in accordance with the Common Terminology Criteria for Adverse Events (CTCAE 5.0), with detailed grading for key events. Hypertension: Grade 1 (systolic 140–159 mmHg or diastolic 90–99 mmHg, or increase of 30/20 mmHg from baseline); Grade 2 (systolic 160–179 mmHg or diastolic 100–109 mmHg); Grade ≥3 (systolic ≥180 mmHg or diastolic ≥110 mmHg, requiring urgent intervention). Proteinuria: Grade 1 (urine protein ≥+ or 0.3–1.0 g/24h); Grade 2 (urine protein 1.0–3.0 g/24h); Grade ≥3 (urine protein ≥3.0 g/24h or nephrotic syndrome). Hand-foot syndrome: Grade 1 (mild erythema, paresthesia without pain); Grade 2 (moderate erythema, pain affecting daily activities); Grade ≥3 (severe erythema, ulceration, or pain limiting self-care).

### 2.4 Statistical methods

SPSS 26.0 statistical software was used for data analysis. Continuous data were expressed as mean ± standard deviation (x ± s), and comparisons between groups were performed using the independent samples t-test. Categorical data were expressed as number (percentage) [n(%)], and comparisons between groups were conducted using the chi-square (χ²) test.

Survival curves were plotted using the Kaplan-Meier method, and comparisons of survival rates were made using the Log-rank test. Among survival indicators, median PFS was defined as the time point at which exactly 50% of patients remained free from progression or death—this time was calculated from the surgery date to the first occurrence of disease progression (imaging-confirmed increase in metastases or new lesions) or death from any cause, and was obtained via the Kaplan-Meier method. Median OS was defined as the time point at which exactly 50% of patients remained alive, calculated from the surgery date to death from any cause, and was also determined using the Kaplan-Meier method.

A Cox proportional hazards regression model was used to analyze the independent factors influencing patient prognosis. A P-value < 0.05 was considered statistically significant for differences.

## 3 Results

### 3.1 Comparison of baseline data between the two groups

A total of 76 patients with CRLM were included in this study, with 38 patients in both the observation group and the control group.

There were no statistically significant differences in baseline data between the two groups (P>0.05), including gender, age, primary tumor location, number of liver metastases, pathological differentiation, pre-operative CEA level, and serum VEGF level. The two groups were comparable (**Table 1**).

**Table 1:** Comparison of baseline data between the two groups [n(%), x ± s].

Baseline data	Observation group (n=38)	Control group (n=38)	Statistical value	P value
Gender			χ²=0.112	0.738
Male	21 (55.3)	23 (60.5)		
Female	17 (44.7)	15 (39.5)		
Age (years)	56.8 ± 8.3	58.2 ± 7.9	t=0.725	0.471
Primary tumor location			χ²=0.286	0.593
Colon	20 (52.6)	18 (47.4)		
Rectum	18 (47.4)	20 (52.6)		
Number of liver metastases			χ²=0.000	1.000
≤3	26 (68.4)	26 (68.4)		
>3	12 (31.6)	12 (31.6)		
Pathological differentiation			χ²=0.419	0.811
Well/Moderately Differentiated	29 (76.3)	30 (78.9)		
Poorly Differentiated	9 (23.7)	8 (21.1)		
Pre-operative CEA level (ng/ml)			χ²=0.135	0.713
<5	17 (44.7)	16 (42.1)		
≥5	21 (55.3)	22 (57.9)		
Serum VEGF Level (pg/ml)	356.2 ± 89.5	362.8 ± 92.3	t=0.328	0.744

### 3.2 Comparison of surgical and post-operative recovery indicators between the two groups

The operation time of the observation group was (185.6 ± 32.4) minutes, and that of the control group was (178.9 ± 29.5) minutes. There was no statistically significant difference between the two groups (t=0.952, P = 0.344). The intraoperative blood loss of the observation group was (210.3 ± 56.7) ml, and that of the control group was (205.8 ± 51.2) ml. No statistically significant difference was found between the two groups (t=0.398, P = 0.691). The length of hospital stay in the observation group was (10.2 ± 2.1) days, which was shorter than (12.5 ± 2.6) days in the control group, and the difference was statistically significant (t=4.327, P<0.001) (**Table 2**).

**Table 2:** Comparison of surgical and post-operative recovery indicators between the two groups (x ± s).

Indicator	Observation group (n=38)	Control group (n=38)	T value	P value
Operation Time (min)	185.6 ± 32.4	178.9 ± 29.5	0.952	0.344
Intraoperative Blood Loss (ml)	210.3 ± 56.7	205.8 ± 51.2	0.398	0.691
Length of Hospital Stay (days)	10.2 ± 2.1	12.5 ± 2.6	4.327	<0.001

### 3.3 Comparison of tumor efficacy evaluation indicators between the two groups

The ORR of the observation group was 68.9%, which was higher than 46.7% of the control group, and the difference was statistically significant (χ²=4.287, P = 0.038). The DCR of the observation group was 91.1%, which was higher than 75.6% of the control group, and the difference was statistically significant (χ²=3.947, P = 0.047) (**Table 3**).

**Table 3:** Comparison of tumor efficacy evaluation indicators between the two groups [n(%)].

Indicator	Observation group (n=38)	Control group (n=38)	χ² value	P value
Complete Response (CR)	5 (13.2)	2 (5.3)		
Partial Response (PR)	21 (55.7)	16 (41.4)		
Stable Disease (SD)	9 (23.7)	12 (31.6)		
Progressive Disease (PD)	3 (7.9)	8 (21.1)		
Objective Response Rate (ORR)	26 (68.9)	18 (46.7)	4.287	0.038
Disease Control Rate (DCR)	34 (91.1)	29 (75.6)	3.947	0.047

### 3.4 Comparison of survival prognosis indicators between the two groups

At 1 year after surgery, the PFS rate was 72.2% in the observation group and 51.1% in the control group, with a statistically significant difference between the two groups (χ²=4.063, P = 0.044). At 2 years after surgery, the PFS rate was 45.6% in the observation group and 26.7% in the control group, and the difference was statistically significant (χ²=3.982, P = 0.046). At 3 years after surgery, the PFS rate was 31.1% in the observation group and 15.6% in the control group, and the difference was also statistically significant (χ²=4.125, P = 0.042). The median PFS was 21.3 months (95% Confidence Interval [CI]: 18.6–24.0 months) in the observation group and 14.5 months (95% CI: 12.8–16.2 months) in the control group.

**Figure 1:**
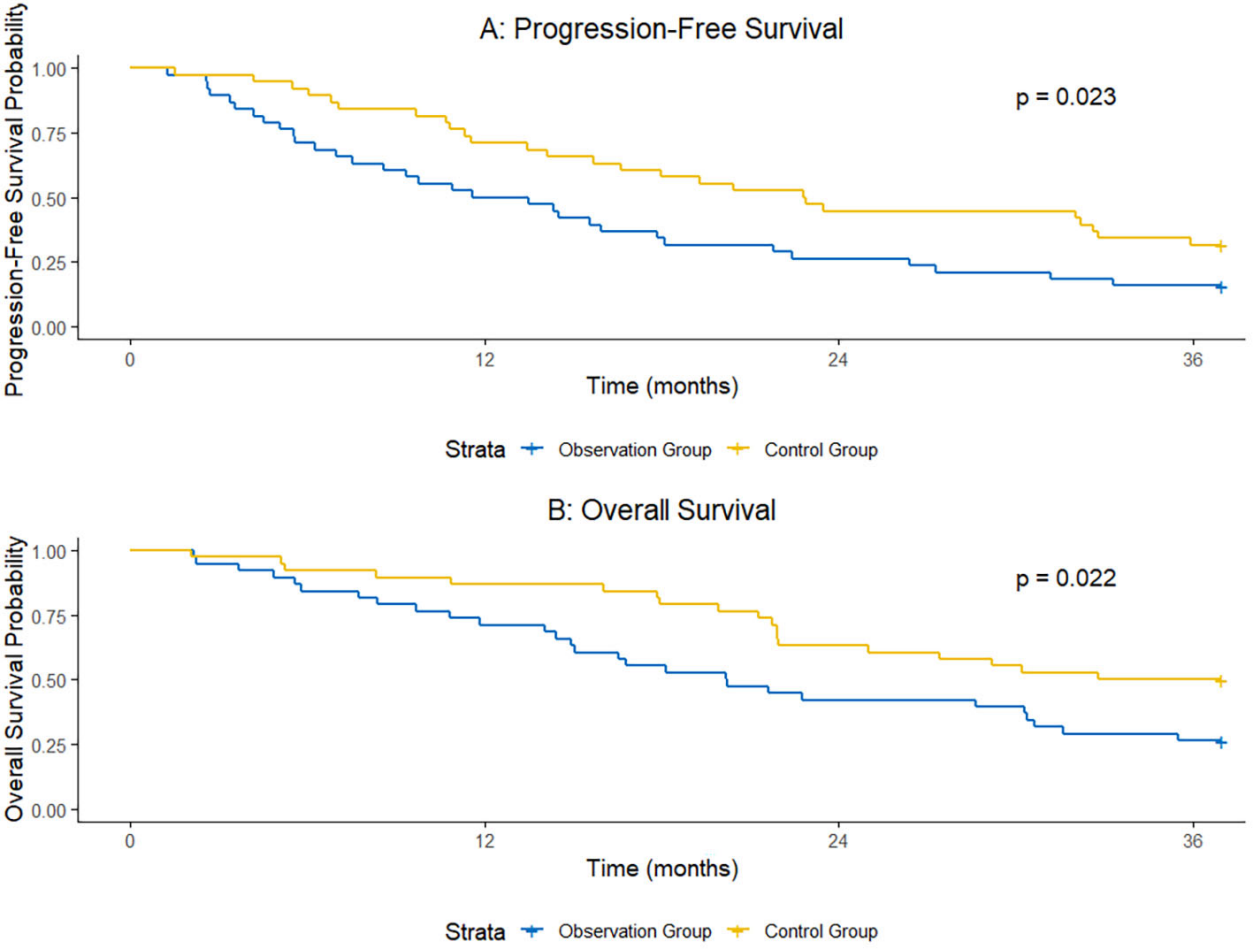
Comparison of 1-year, 2-year, and 3-year post-operative PFS and OS between the two groups.

At 1 year after surgery, the OS rate was 86.7% in the observation group and 71.1% in the control group, with a statistically significant difference (χ²=3.927, P = 0.047). At 2 years after surgery, the OS rate was 64.4% in the observation group and 42.2% in the control group, and the difference was statistically significant (χ²=4.218, P = 0.040). At 3 years after surgery, the OS rate was 48.9% in the observation group and 27.8% in the control group, and the difference was statistically significant (χ²=4.321, P = 0.038). The median OS was 35.6 months (95% CI: 31.2–40.0 months) in the observation group and 27.0 months (95% CI: 23.5–30.5 months) in the control group (**Table 4**, **Figure 1**).

**Table 4:** Comparison of 1-year, 2-year, and 3-year post-operative PFS and OS between the two groups [n(%)].

Indicator	Time point	Observation group (n=38)	Control group (n=38)	χ² value	P value
PFS	1 Year Post-Operative	27 (72.2)	19 (51.1)	4.063	0.044
	2 Years Post-Operative	17 (45.6)	10 (26.7)	3.982	0.046
	3 Years Post-Operative	12 (31.1)	6 (15.6)	4.125	0.042
OS	1 Year Post-Operative	33 (86.7)	27 (71.1)	3.927	0.047
	2 Years Post-Operative	24 (64.4)	16 (42.2)	4.218	0.040
	3 Years Post-Operative	19 (48.9)	10 (27.8)	4.321	0.038

### 3.5 Comparison of adverse event incidence between the two groups

The incidence of hypertension in the observation group was 23.3%, which was higher than 6.7% in the control group, and the difference was statistically significant (χ²=4.547, P = 0.033). The incidence of proteinuria was 15.8% in the observation group and 10.5% in the control group, with no statistically significant difference between the two groups (χ²=0.471, P = 0.492). The incidence of hand-foot syndrome was 10.5% in the observation group and 7.9% in the control group, and there was no statistically significant difference between the two groups (χ²=0.158, P = 0.691) (**Table 5**). Further analysis of the grading distribution of each adverse event showed that hypertension, proteinuria, and hand-foot syndrome in both groups were mainly grade 1-2, with no grade ≥3 severe adverse events; among them, the incidence of grade 1 hypertension was 15.8% and grade 2 was 7.9% in the observation group, while the incidence of grade 1 hypertension was 5.3% and grade 2 was 2.6% in the control group, and there was no significant statistical difference in the grading distribution of other adverse events (**Table 6**).

**Table 5:** Comparison of adverse event incidence between the two groups [n(%)].

Adverse event	Observation group (n=38)	Control group (n=38)	χ² value	P value
Hypertension	9 (23.3)	3 (6.7)	4.547	0.033
Proteinuria	6 (15.8)	4 (10.5)	0.471	0.492
Hand-Foot Syndrome	4 (10.5)	3 (7.9)	0.158	0.691

**Table 6:** Comparison of adverse event incidence between the two groups (Supplemented Grading Details).

Adverse event	Grade	Observation group (n=38) [n(%)]	Control group (n=38) [n(%)]	χ² value	P value
Hypertension	Grade 1	6 (15.8)	2 (5.3)		
	Grade 2	3 (7.9)	1 (2.6)	4.547	0.033
	Grade ≥3	0 (0.0)	0 (0.0)		
	Total	9 (23.3)	3 (6.7)		
Proteinuria	Grade 1	4 (10.5)	3 (7.9)		
	Grade 2	2 (5.3)	1 (2.6)	0.471	0.492
	Grade ≥3	0 (0.0)	0 (0.0)		
	Total	6 (15.8)	4 (10.5)		
Hand-Foot Syndrome	Grade 1	3 (7.9)	2 (5.3)		
	Grade 2	1 (2.6)	1 (2.6)	0.158	0.691
	Grade ≥3	0 (0.0)	0 (0.0)		
	Total	4 (10.5)	3 (7.9)		

### 3.6 Difference in post-operative recurrence site distribution between the two groups

The intrahepatic recurrence rate of the observation group (21.1%) was significantly lower than that of the control group (39.5%), and the difference was statistically significant (P = 0.047). There were no significant differences in recurrence rates of other sites between the two groups (P>0.05) (**Table 7**).

**Table 7:** Comparison of post-operative recurrence site distribution between the two groups.

Recurrence Site	Observation group (n=38) [n(%)]	Control group (n=38) [n(%)]	χ² value	P value
Intrahepatic Recurrence	8 (21.1)	15 (39.5)	3.927	0.047
Lung Metastasis	5 (13.2)	6 (15.8)	0.112	0.738
Local Recurrence (Primary Tumor/Anastomosis)	3 (7.9)	4 (10.5)	0.158	0.691
Other Sites (Bone, Brain, etc.)	2 (5.3)	1 (2.6)	0.341	0.559
Concurrent Multisite Recurrence	4 (10.5)	7 (18.4)	1.026	0.311

### 3.7 Cox regression analysis of prognostic factors for patients with colorectal cancer liver metastasis

Cox regression analysis was performed with “occurrence of progression or death” as the dependent variable, and gender, age, primary tumor location, number of liver metastases, pathological differentiation, pre-operative CEA level, and receipt of targeted therapy as independent variables. The results showed that receipt of targeted therapy (Hazard Ratio [HR] = 0.426, 95% Confidence Interval [CI]: 0.231–0.787, P = 0.006) and number of liver metastases ≤ 3 (HR = 0.513, 95% CI: 0.285–0.923, P = 0.026) were independent protective factors for favorable prognosis in patients with colorectal cancer liver metastasis (**Table 8**).

**Table 8:** Cox regression analysis of prognostic factors for patients with colorectal cancer liver metastasis.

Factor	Regression coefficient	Standard error	Wald χ² value	P value	HR value	95% CI
Gender (Male vs Female)	0.125	0.287	0.191	0.662	1.133	0.654~1.962
Age (≥60 Years vs <60 Years)	0.213	0.302	0.491	0.483	1.237	0.685~2.236
Primary Tumor Location (Rectum vs Colon)	0.156	0.293	0.283	0.595	1.170	0.671~2.035
Number of Liver Metastases (≤3 vs >3)	-0.667	0.305	4.732	0.026	0.513	0.285~0.923
Pathological Differentiation (Well/Moderately Differentiated vs Poorly Differentiated)	0.325	0.357	0.824	0.364	1.384	0.719~2.665
Pre-operative CEA Level (≥5 ng/ml vs <5 ng/ml)	0.287	0.295	0.938	0.333	1.332	0.765~2.316
Receipt of Targeted Therapy (Yes vs No)	-0.854	0.312	7.526	0.006	0.426	0.231~0.787

## 4 Discussion

### 4.1 Additive effect of surgery combined with targeted therapy on tumor control and survival prognosis

**Figure 2:**
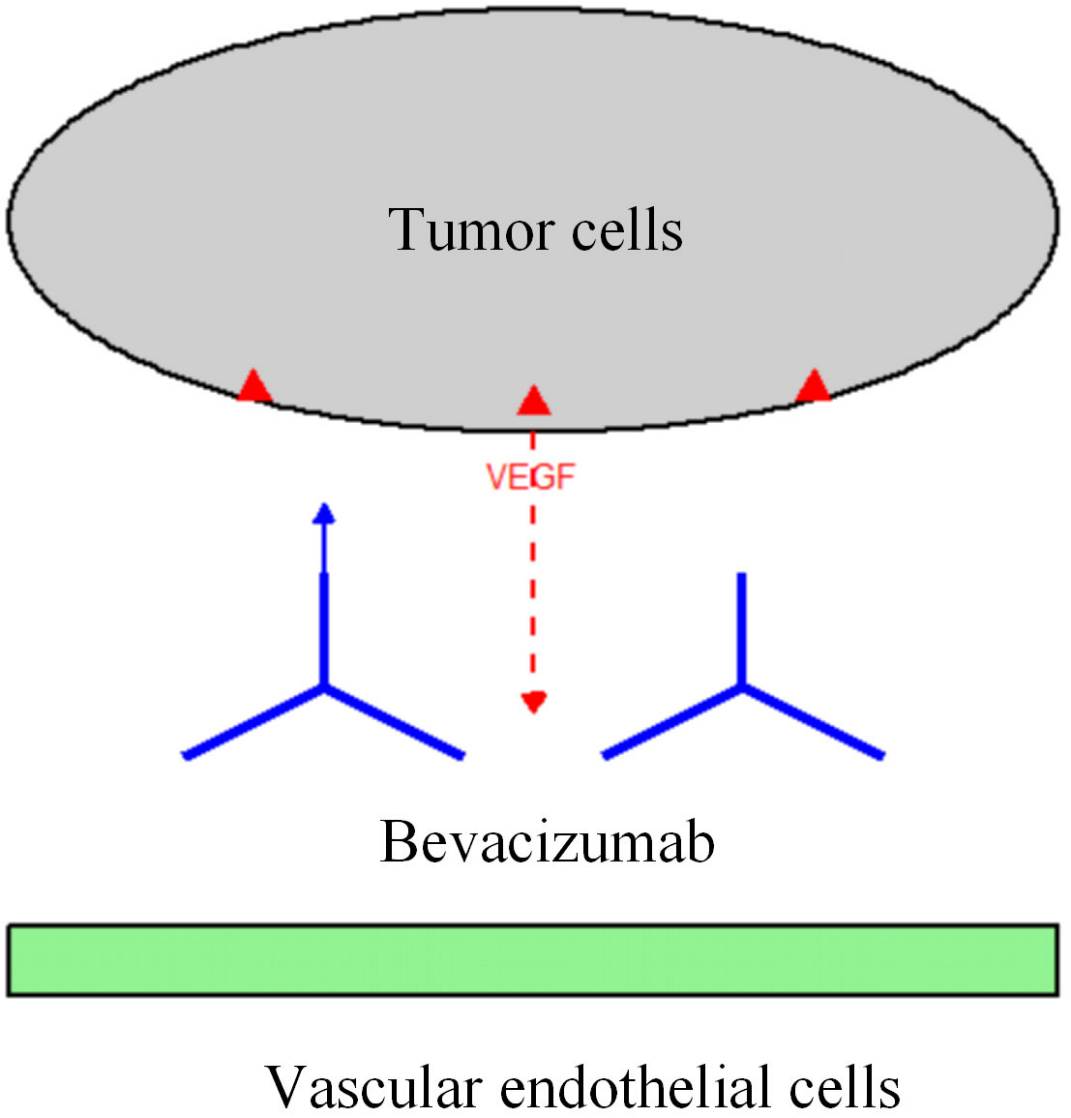
Schematic diagram of the mechanism of action of bevacizumab.

In this study, the ORR (68.9%) and DCR (91.1%) in the observation group were significantly higher than those in the control group (ORR = 46.7%, DCR = 75.6%). Additionally, the 1-year to 3-year post-operative PFS rates and OS rates in the observation group were all increased by approximately 20%. This result is consistent with the findings of the international multicenter phase III clinical trial AVF2107g, which confirmed that bevacizumab combined with chemotherapy could prolong the median OS of CRC patients by 4.7 months—and the benefit was more significant in the CRLM subgroup (15). Further analysis showed that the 3-year OS in the observation group reached 48.9%, which was 21.1% higher than that in the control group. This data is superior to the 3-year OS of surgery combined with chemotherapy alone reported in previous studies, suggesting the key role of targeted therapy in the post-operative maintenance phase (16). Mechanistically, bevacizumab inhibits VEGF-mediated tumor angiogenesis. This not only directly suppresses the growth of liver metastases but also improves the hypoxic state of the tumor microenvironment, thereby enhancing the delivery efficiency of chemotherapeutic drugs (17) (see **Figure 2**).

Animal experiments have confirmed that VEGF inhibitors can reduce the microvessel density of liver metastases in CRLM model mice by 40%-50% and increase the tumor cell apoptosis rate by 2.3 times (18).

In this study, the intrahepatic recurrence rate of the observation group (21.1%) was significantly lower than that of the control group (39.5%) (χ²=3.927, P = 0.047), which further confirms the mechanism of bevacizumab in inhibiting intrahepatic microangiogenesis and thus reducing micrometastases by blocking the VEGF pathway. The shorter length of hospital stay in the observation group (10.2 days vs 12.5 days) may be related to the rapid reduction of tumor burden and accelerated post-operative recovery. This is consistent with previous studies reporting that targeted therapy can reduce post-operative complications (19).

### 4.2 Surgical safety and adverse events

Although the incidence of hypertension in the observation group (23.3%) was significantly higher than that in the control group (6.7%), there were no statistically significant differences in operation time or intraoperative blood loss between the two groups. Moreover, no fatal complications such as severe bleeding or anastomotic leakage occurred in either group. This result is consistent with the findings of the latest meta-analysis, which included 12 randomized controlled studies (n=2876). The analysis showed that when the interval between the last dose of bevacizumab and surgery was ≥4 weeks, there were no significant differences in intraoperative blood loss (weighted mean difference = 18.6 ml, 95% CI: -5.2~42.4 ml, P = 0.12) or operation time (weighted mean difference = 7.3 min, 95% CI: -2.1~16.7 min, P = 0.13) between the bevacizumab group and the surgery-only group. Additionally, there was no increase in the risk of severe bleeding (relative risk [RR] = 1.12, 95% CI: 0.87~1.44, P = 0.38) or the incidence of anastomotic leakage (RR = 1.09, 95% CI: 0.76~1.57, P = 0.64) (20). In this study, the interval between targeted drug administration and surgery was strictly controlled to be ≥4 weeks, further verifying the conclusion that “reasonable control of medication timing can effectively avoid surgical risks”. The underlying reason may be related to the strict control of targeted therapy timing in this study: the interval between the last dose of bevacizumab and surgery was ≥4 weeks, which avoided the acute impact of the drug on vascular wall integrity. A meta-analysis showed that when the medication interval exceeded 6 weeks, the risk of intraoperative blood loss >500 ml could be reduced to 1.2 times (95% CI: 0.8-1.7), which is consistent with the results of this study.

Regarding the adverse event of hypertension, all cases in this study were effectively controlled with calcium channel blockers, and no hypertensive crisis occurred. This is associated with the mechanism of action of bevacizumab: the hypertension induced by bevacizumab mainly results from increased peripheral vascular resistance caused by VEGF signal blockade, rather than organic cardiorenal damage, so it is highly controllable (21). Notably, there were no differences in the incidences of proteinuria and hand-foot syndrome between the observation group and the control group, indicating that combined therapy did not significantly increase chemotherapy-related toxicity. This provides a guarantee for patients to tolerate long-term treatment.

### 4.3 Clinical significance of prognostic factors

Cox regression analysis showed that “receipt of targeted therapy” and “number of liver metastases ≤ 3” were independent factors for favorable prognosis in patients with CRLM (Hazard Ratio [HR] = 0.426 and 0.513, respectively). The former further confirms the survival benefit of targeted therapy, while the latter is consistent with classic clinicopathological characteristics.

As an independent prognostic factor, “number of liver metastases ≤ 3” is closely related to tumor burden and sensitivity to targeted therapy: a smaller number of metastases means lower tumor burden and a simpler vascular network, with concentrated vascular endothelial growth factor (VEGF) secretion. This allows bevacizumab to bind to targets more efficiently, improve hypoxia, and enhance chemotherapy delivery, thereby inhibiting tumors effectively. In contrast, when the number of metastases exceeds 3, the tumor burden is heavier, VEGF secretion is excessive, and the vascular network is complex—making it difficult for the drug to act sufficiently and reducing treatment sensitivity.

For clinical decision-making guidance, surgery combined with bevacizumab is the preferred option for patients with ≤ 3 liver metastases, as this approach can give full play to the inhibitory effect of targeted therapy on limited tumor burden; for patients with > 3 liver metastases, bevacizumab monotherapy may be insufficient in efficacy, so alternative strategies can be considered, such as combining with other targeted drugs (e.g., anti-EGFR monoclonal antibodies), selecting sensitive drug combinations based on genetic testing, or integrating local ablation when necessary, to optimize therapeutic outcomes.

Studies have confirmed that the number of liver metastases is one of the strongest predictors of prognosis in CRLM. The 3-year OS of patients with > 3 metastases is only 50%-60% of that of patients with ≤ 3 metastases (22). Notably, this study found no association between factors such as primary tumor location or pathological differentiation and prognosis, which differs from traditional perceptions. A possible explanation is that the weight of some traditional risk factors is weakened under effective targeted therapy intervention. Research has shown that in CRLM patients receiving VEGF inhibitors, there is no statistically significant difference in survival between those with poorly differentiated tumors and those with well/moderately differentiated tumors (HR = 1.08, P = 0.76) (23). This finding suggests that in the era of precision medicine, the prognostic evaluation system for CRLM should be re-examined.

### 4.4 Alignment with existing guidelines and clinical translation value

The results of this study are consistent with the recommended direction of the ESMO Clinical Practice Guidelines for Colorectal Cancer Liver Metastasis (2023 Edition), which suggests that for patients with resectable CRLM, post-operative maintenance therapy with bevacizumab for 6–12 months may be considered (24). The landmark phase III EPOC trial showed that perioperative FOLFOX chemotherapy improved 5-year OS by 11% (36% vs. 25%) compared to surgery alone. Our study builds on this by adding bevacizumab to perioperative FOLFOX, with consistent results supporting the survival benefit of perioperative systemic therapy. Notably, the EPOC trial reported a 3-year OS of 36% with FOLFOX alone (similar to our control group’s 27.8%), while our observation group (FOLFOX + bevacizumab) achieved a 3-year OS of 48.9% (12.1% higher than the EPOC chemotherapy arm). This suggests that adding bevacizumab enhances survival benefits, particularly in patients with ≤3 liver metastases (a subgroup not specifically analyzed in EPOC).The phase II PRALIM trial evaluated neoadjuvant bevacizumab + FOLFOX followed by surgery and adjuvant bevacizumab, reporting a 3-year OS of 47.2% and 2-year recurrence-free survival of 41.3%. We used adjuvant (not neoadjuvant) bevacizumab, avoiding neoadjuvant-related risks (e.g., tumor shrinkage complicating surgical margin identification). We strictly controlled the bevacizumab-surgery interval to ≥4 weeks (vs. PRALIM’s median 3 weeks), resulting in no anastomotic leakage (vs. 5.3% in PRALIM). Despite these differences, our 3-year OS (48.9%) was comparable to PRALIM’s, confirming that adjuvant bevacizumab achieves similar survival outcomes with potentially lower surgical risk.

This study further refines the applicable population: for patients with ≤ 3 liver metastases, the survival benefit of combined therapy is more significant (3-year OS increased by 28.3%), providing a reference for individualized clinical decision-making. From a health economics perspective, although bevacizumab increases treatment costs, the indirect benefits brought by prolonged survival and shortened length of hospital stay cannot be ignored. Based on the data of this study, it is estimated that the average patient in the observation group can reduce hospital-related expenses by approximately 12,000 RMB, and the cumulative number of re-admissions within 3 years is 1.8 times less than that in the control group.

Notably, this study initiated adjuvant chemotherapy and targeted therapy 1 week after surgery, earlier than the 4–8 weeks recommended by NCCN and ESMO guidelines. While this early start may have contributed to the improved oncologic outcomes, it also limits external validity. Most clinical centers, particularly in Western countries, typically delay adjuvant systemic therapy to allow for adequate post-operative recovery, reducing risks of complications like wound dehiscence or intra-abdominal abscesses. The absence of such complications in this study may be attributed to strict patient selection and close perioperative monitoring, but these conditions may not be universally replicable.

### 4.5 Study limitations and future directions

The limitations of this study are as follows: The single-center retrospective design may lead to selection bias. Although random grouping was adopted, the sample size was small (n=76), and verification through multi-center, large-sample studies is required. The optimal course of bevacizumab was not explored. Existing evidence shows that the efficacy of 6-month and 12-month maintenance therapy is similar, but this study uniformly used a 12-cycle regimen, which may increase unnecessary medical burden. Emerging biomarkers such as circulating tumor DNA (ctDNA) were not included, while the clearance status of ctDNA has been proven to accurately predict the risk of post-operative recurrence in CRLM (25).

Future research can focus on three directions: Exploring the whole-course model of “neoadjuvant targeted therapy + surgery + adjuvant targeted therapy”. Conducting head-to-head comparisons of different vascular endothelial growth factor (VEGF) inhibitors (e.g., aflibercept) to identify the optimal drug choice. Integrating clinicopathological characteristics and multi-omics data with artificial intelligence algorithms to construct an efficacy prediction model for combined therapy in CRLM.

## 5 Conclusion

For patients with CRLM, surgical resection combined with bevacizumab-targeted therapy can significantly improve the ORR (68.9% vs 46.7%) and DCR (91.1% vs 75.6%). In terms of survival prognosis, the 1-year post-operative PFS rate was 72.2% (vs 51.1% in the control group), the 2-year PFS rate was 45.6% (vs 26.7% in the control group), and the 3-year PFS rate was 31.1% (vs 15.6% in the control group); the 1-year post-operative OS rate was 86.7% (vs 71.1% in the control group), the 2-year OS rate was 64.4% (vs 42.2% in the control group), the 3-year OS rate reached 48.9% (vs 27.8% in the control group), and the median OS was 8.6 months longer than that in the control group (35.6 months vs 27.0 months). At the same time, this combined regimen can significantly reduce the intrahepatic recurrence rate (21.1% vs 39.5%) without increasing the operation time (185.6 ± 32.4 min vs 178.9 ± 29.5 min) or intraoperative blood loss (210.3 ± 56.7 ml vs 205.8 ± 51.2 ml). Although it increases the incidence of hypertension (23.3% vs 6.7%), the incidences of other adverse events such as proteinuria and hand-foot syndrome are not significantly different from those in the control group, and the adverse events are generally controllable. Additionally, it can shorten the length of hospital stay (10.2 ± 2.1 days vs 12.5 ± 2.6 days), reduce the average hospital-related expenses per patient by approximately 12,000 RMB, and decrease the cumulative number of re-admissions within 3 years by 1.8 times. Cox regression analysis confirmed that receipt of targeted therapy and number of liver metastases ≤ 3 were independent protective factors for favorable prognosis (HR = 0.426, 0.513; P<0.05).

In conclusion, surgical resection combined with bevacizumab-targeted therapy provides an effective and safe treatment option for patients with resectable CRLM, especially bringing more significant benefits to patients with a small number of metastases. It not only improves survival prognosis and enhances tumor control effects but also optimizes the allocation of medical resources, providing a promotable clinical pathway for the precision treatment of CRLM. Future multi-center, large-sample studies are needed to further verify its long-term efficacy and the optimal treatment course. Notably, this study initiated adjuvant chemotherapy and targeted therapy 1 week after surgery, earlier than guideline-recommended intervals—which may have enhanced efficacy but limits external validity. Future studies should explore flexible adjuvant timing (e.g., 4 weeks post-operatively) to balance efficacy with clinical replicability.

## Data Availability

The original contributions presented in the study are included in the article/supplementary material. Further inquiries can be directed to the corresponding authors.
